# Supplementary material for: Continental-scale genomic surveillance of Plasmodium falciparum malaria across sub-Saharan Africa with rapid nanopore sequencing
Source: Nat Commun. 2026 May 11;17:4218. doi: 10.1038/s41467-026-72358-z (PMC13161212; doi:10.1038/s41467-026-72358-z)
Supplement: Supplementary file 1 — Supplementary Information [file 41467_2026_72358_MOESM1_ESM.pdf]

Supplementary Information

Continental-scale genomic surveillance of *Plasmodium falciparum* malaria across sub-Saharan Africa with rapid nanopore sequencing

Mulenga Mwenda, Karolina Mosler, Bernd Bohmeier, Miriam Chomba, Welmoed Van Loon, Brenda Mambwe, Amy Gaye, Adedolapo Olorunfemi, Salma Suliman, Nassandba Julien Yanogo, Djiby Sow, Bassirou Ngom, Oumou Aïcha Zeïna Zoure, Yssimini Nadège Guillène Tibiri, Fiyinfoluwa Ojeniyi, Arsène Zongo, Etilé A. Anoh, Vincent Achi, Carol Chiyesu, Sheila Otieno, Bixa Ogola, Moussa Niangaly, Manuela Carrasquilla, Dagaga Kenea Goboto, Torsten Feldt, Tafese Beyene Tufa, Rafael Oliveira, Emma Schallenberg, Yuhana Sogoba, Christina Ntalla, Oumarou Ouedraogo, Kephas Otieno, Oluyinka Opaleye, Adekunle Olowe, Marley Gibbons, Chris Drakeley, Grit Schubert, Frank P. Mockenhaupt, Silvia Portugal, Awa B. Deme, Issiaka Soulama, Daouda Ndiaye, Olusola Ojurongbe, Simon Kariuki, Ya Ping Shi, Jonathan S. Schultz, Moonga Hawela, Daniel J. Bridges, Jason A. Hendry

Contents

1 Supplementary Notes **1**

2 Supplementary Tables **4**

3 Supplementary Figures **9**

1 Supplementary Notes

Supplementary Note 1. We estimated the approximate number of samples required to reliably detect if a mutation exceeded ∼1-2% prevalence anywhere across malaria-endemic Africa, as follows. Let our goal be to detect the presence of a mutation with prevalence p at a given collection site, with at least 80% power. Assuming random independent sampling, the probability of detecting the mutation in at least one sample, which is equivalent to the power, is $1-(1-p)^n$, where n is the number of samples sequenced. From this formula, we can calculate that with 80% power: $n = 161$ when $p = 0.01$ and $n = 80$ when $p = 0.02$. There are 44 malaria-endemic countries in Africa and we posit that an average of 15 collection sites per country would provide reasonable geographical coverage (some countries e.g. Nigeria will require more and some e.g. Burundi, less). Then it would be required to sequence 106,260 (= 44 x 15 x 161) to 52,800 (= 44 x 15 x 80) samples to detect emerging mutations at 1 to 2% prevalence, respectively.

Supplementary Note 2. The observed median WSAF across heterozygous SNPs reflects the mock DBS minor clone proportion, while variation around the expected WSAF is introduced primarily by molecular variability during DBS processing (i.e. DNA extraction, multiplex PCR, and library preparation), as well as potential sequencing error. Consistent with this, at 100 parasites/μL we observed greater WSAF deviations than at higher parasitemia levels (100 parasites/μL: root mean squared error [RMSE] 7.0%, $n = 1,127$

heterozygous SNPs; 1,000 parasites/μL: RMSE 4.0%, $n = 1,128$; 10,000 parasites/μL: RMSE 3.3%, $n = 1,127$). Moreover, we found that haplotypes carried by the 5% and 2.5% minor clones were often absent at 100 parasites/μL: in 39.6% (25/63 amplicons) and 55.0% (33/60 amplicons) of cases, respectively, they produced no WSAF signal (Supplementary Fig. 6). However, effectively all haplotypes with a minor clone proportion of 10% at 100 parasites/μL generated signal (98.3%, 59/60 amplicons). This suggests that, regardless of the subsequent bioinformatics analysis, the NOMADS-MVP protocol has a minimum minor clone LoD of above approximately 5 parasites/μL (5% of 100 parasites/μL), set by the molecular stochasticity and the sensitivity of the laboratory steps. Achieving a minor clone LoD below this would require, for example, using more efficient DNA extraction protocols or developing more sensitive PCRs.

Supplementary Note 3. For all false-negatives in which a true heterozygous SNP was incorrectly called homozygous reference, the WSAFs were also very low (median 0.1%, IQR 0.0–1.3%, max 3.7%, $n = 422$); conversely, for false-negatives in which a true heterozygous SNP was called as homozygous alternative, the WSAFs were very high (median 99.9%, IQR 99.1–100%, min 98.1%, $n = 80$; Fig. 3c). This suggests that the majority of false-negatives are caused by molecular stochasticity and sensitivity of the NOMADS-MVP protocol (as discussed in Supplementary Note 2), rather than by *Delve*.

Supplementary Note 4. Antimalarial resistance marker identification is central to genomic surveillance. Therefore, we specifically investigated the recovery of WHO-defined antimalarial resistance markers across the described mock DBS samples, as well as in mock samples created from three Cambodian *P. falciparum* strains carrying validated artemisinin resistance mutations (*kelch13* R539T, I543T, and C580Y), and sequenced in duplicate. In clonal mock samples ($n = 33$), *Delve* identified all antimalarial resistance markers with 100% accuracy (no false-positives or false-negatives; Supplementary Table 4). *Delve* also identified all resistance markers with 100% accuracy in polyclonal mock DBS samples with 1000 parasites/μL or greater down to a minor clone frequency of 5%, except for *mdr1* N86Y (accuracy 77.8%; Supplementary Table 4). Because the *mdr1* gene is present in two or three copies in our *Dd2* clone, the N86Y marker WSAF is typically less than half the minor clone frequency, making the mutation harder to detect and explaining the reduced accuracy. At 100 parasites/μL, *Delve* identified antimalarial resistance markers with 98.8% and 92.2% accuracy for polyclonal mock DBS samples with minor clones at 20% and 10% frequency, respectively (Supplementary Table 4). Importantly, no false-positive SNP impacted any codon associated with antimalarial drug resistance; the observed reductions in accuracy were exclusively due to false-negatives arising when markers carried by minor clones fell below the LoD.

Supplementary Note 5. The few examples where mean precision noticeably declined in Figure 3d were caused by the clonal 3D7 mock DBS samples (e.g. 150× mean coverage at 10,000 parasites/μL: 93.4% including 3D7 [$n = 27$] versus 99.6% excluding 3D7 [$n = 18$]); 3D7 has only 1 true positive SNP, and so a single false-positive reduces the precision to 50% and substantially depresses the mean.

Supplementary Note 6. The two discrepancies included one sample that was *hrp2*−/*hrp3*− by NOMADS-MVP but *hrp2*+/*hrp3*− by conventional PCR (Fig. 4c, indicated by 'ii'). This sample had a mean coverage of 425× over the *hrp2* amplicon; a much higher mean coverage than other samples predicted as *hrp2*− (median 0.3×, IQR 0.0–0.9×), but lower than those predicted *hrp2*+ (median 1,468×, IQR 934–3,433). Heterozygosity over *ama1* ($H = 0.085$) indicated that the sample was a mixed infection with at least two *P.f.* clones. A possible explanation for this discrepancy is that one clone carries an *hrp2* deletion that is detected by the statistical model, but still yields a positive result by conventional PCR. Similarly, the second discrepancy (*hrp2*−/*hrp3*− by NOMADS-MVP, *hrp2*−/*hrp3*+ by conventional PCR) had non-negligible coverage over *hrp3* (78×) and *ama1* heterozygosity suggesting a mixed infection ($H = 0.049$; Fig. 4c, indicated by 'iii'). We also noted one other sample where, although conventional PCR and NOMADS-MVP both predicted an *hrp3* deletion, considerable coverage over the *hrp3* (479×) was present (Fig. 4c, indicated by 'i'). None of the discordant samples had particularly low parasitemia (i: 2,960 parasites/μL; ii: 4,960 parasites/μL; iii: 9,080 parasites/μL) or mean coverage (i: 1,505×; ii: 1,752×; iii: 3,753×), which might be expected if insufficient sensitivity was an underlying cause. All samples whose parasitemia or mean coverage was in the lowest decile (229–960 parasites/μL; or 323–647×) had corresponding *hrp2/3* deletion calls between the PCR-based assays and NOMADS-MVP.

Supplementary Note 7. The Rapid Barcoding Kit (SQK-RBK114.96) from ONT leverages transposome-based chemistry that cleaves sample DNA during barcode ligation. The barcoding reaction takes just four minutes, but the sample DNA is fragmented in the process. To characterise the impact on read lengths, we randomly selected 80 samples from across four different sequencing experiments, including field DBS samples from Kenya ($n = 20$), Ethiopia ($n = 20$) and Mali ($n = 20$), as well as a set of mock DBS samples created from laboratory strains in Germany ($n = 20$). Across these samples, a total of 3.45 million reads mapped uniquely to the *P. falciparum* reference genome and their median length was 567 bp (IQR 376–808 bp). There was considerably more read length variation within individual samples than between samples or experiments (Supplementary Fig. 8a). The consistency across four independent sample sets and experiments suggests that the read length distribution is robust to technical and biological factors. Similarly, in optimisation experiments we found that read lengths were insensitive to variation in the barcoding reaction time or input DNA mass (Supplementary Fig. 9). The within-sample read length variation was driven by the different amplicons in our panel, with, as expected, longer amplicons generating longer reads (Supplementary Fig. 8b). In particular, when grouped by the amplicon to which they mapped, reads had a median length of roughly half of their amplicon length (mean 59%, range 45%–69%), consistent with a single cleavage and barcoding event per amplicon (Supplementary Fig. 8b). Across amplicons, the longest median read length was for the *kelch13* amplicon at 872 bp and the shortest for the *mdr1* N-terminal amplicon at 397 bp.

Longer reads support physical phasing of heterozygous variants and the investigation of repetitive regions, but only when the variants or regions are fully spanned by individual reads. To this end, we examined whether our data contained reads that fully spanned regions of interest for *P. falciparum* genomic surveillance (Supplementary Fig. 8c). All regions investigated had fully-spanning reads at appreciable frequencies across the eighty samples. For example, a median of 29.4% (IQR 26.4%–33.5%) of reads per sample overlapping the Kelch-repeat propeller domain fully spanned the region. For *dhps*, which mediates Sulfadoxine resistance, a median of 40.0% (IQR 35.9–43.0%) of reads spanned from codon 436 to 613, enabling joint interrogation of the key resistance markers (S436A, A437G, K540E, A581G and A613S). A high-diversity region of *ama1* (LaVerriere et al., 2022) was spanned by a median of 76.5% (IQR 72.1–80.6%) of reads per sample. Finally, key regions of the RTS,S/AS01 and R21/Matrix-M vaccine target *csp* also had fully-spanning reads, including the entire portion used in the vaccine (codons 199–397, median 34.6% of reads fully-spanning) and the entire NANP central repeat region (codons 105–272, median 40.2%).

2 Supplementary Tables

Item	Manufacturer	Catalog #	Pack Size	Unit Cost ($)	Units	Cost ($)
1.5 ml DNA LoBind tubes	Eppendorf	0030 108.051	250 tubes	18.97	1	18.97
100ml reservoir	ThermoFisher	10125707	100 case	248	1	248.00
Adhesive seal sheets	Greiner	676001	100 seals	94	2	188.00
Gloves	Handsafe	GN90-L	2000 gloves	88.01	3	264.03
PCR strip tubes	ThermoFisher	AB2005	2000 wells	354	1	354.00
Plates 96 well (skirted)	ThermoFisher	AB-0800	25 plates	115.94	7	811.58
Qubit Assay Tubes	Invitrogen	Q32856	500 tubes	111.41	5	557.05
Sharpie markers	Stabilo	01-19-2763	10 per pack	15.89	1	15.89
Tips 10ul	–	–	4800 tips	396.29	1	396.29
Tips 200ul	–	–	4800 tips	396.29	1	396.29
Tips 200ul (Multichannel)	–	–	960 tips	192.32	13	2500.16
Tips 20ul	–	–	4800 tips	396.29	1	396.29
Tips 20ul (Multichannel)	–	–	960 tips	192.32	26	5000.32
Tips 1000ul	–	–	3072 tips	358.54	1	358.54
**Total (plasticware)**						**10,948.32**
**Cost per sample (plasticware)**						**5.47** (26.2%)
Agarose	ThermoFisher	16500500	500 gram	675	1	675.00
AMPure XP beads	Beckman Coulter	A63881	60 ml	1485.20	1	1485.20
DNA ladder (1kb)	ThermoFisher	SM0314	100 lanes	64.99	2	129.98
Ethanol	Sigma	E7024-500ML	500 ml	42.61	2	85.22
Flow Cell Priming Kit	ONT	EXP-FLP004	6 rxns	35.04	4	140.16
Flow Cell Wash Kit	ONT	EXP-WSH004	6 washes	99.10	4	396.40
Gel loading dye	NEB	B7024S	4 ml	58.86	1	58.86
KAPA HIFI ReadyMix	Roche	KK2602	6.25 ml	1064.21	6	6385.26
MinION Flow Cell (R10.4.1) 24 pack	ONT	FLO-MIN114	24 flow cell	14400	1	14400.00
Nuclease free water	Invitrogen	AM9937	500 ml	155.19	1	155.19
PCR Primers: MVP	IDT	N/A	3000 rxn	120	1	120.00
Qubit 1x dsDNA HS Assay Kit	ThermoFisher	Q33231	500 rxn	444.32	5	2221.60
Rapid Barcoding Sequencing Kit 96	ONT	SQK-RBK114.96	12 rxn	990.99	4	3963.96
TE buffer (1X) pH 8.0 low EDTA	ThermoFisher	J75793.AP	500 ml	120	1	120.00
**Total (reagents)**						**30,893.88**
**Cost per sample (reagents)**						**15.45** (78.3%)
**Total**						**41,842.00**
**Cost per sample**						**20.92** (100.0%)

Supplementary Table 1: Itemized list of plasticware and reagents required for NOMADS-MVP. 'Units' were calculated to allow for processing 2000 samples. A 10% mastermix overage was included where relevant. We assumed 48 samples/run and 2 runs per flow cell. All costs are in USD and were taken from quotes from Carramore International Limited (`https://www.carramore.com/`) for shipments to Africa made in 2025. No specific manufacturer for pipette tips is recommended, as they should be selected for compatibility with available pipettes. The cost of DNA extraction is not included.

Application	Item	Manufacturer	Catalogue #	Item Cost ($)
DNA quantification	Qubit 4 Fluorometer	ThermoFisher	Q33238	$4,400
Sequencing	MinION	ONT	Mk1D†	$3,000
Process data	Laptop with 1 Tb SSD, Nvidia GPU (16Gb RAM), i7 or equivalent processor, 16 Gb RAM	-	-	$3,000
Store data	5 Tb Storage Drive	-	-	$200
DNA clean-up	Plate Magnet	ThermoFisher	12027	$1,100
DNA clean-up	Tube Magnet	ThermoFisher	12321D	$800
**Total**				**$12,500**

Supplementary Table 2: Itemized list of equipment required for NOMADS-MVP. Main equipment for NOMADS-MVP is shown, basic laboratory equipment (PCR machine, vortex, centrifuge) is excluded. †The Mk1D will soon replace the Mk1B; note this cost includes a package of starting reagents. All costs are in USD and were taken from Carramore International Limited (`https://www.carramore.com/`). No specific recommendations are made for the laptop or external hard drive.

Protocol Name	Platform	Incubation Time (mins)	Pipette Steps	Per-sample cost (USD)	Reference
MAD^{4}HatTeR	Illumina	205	404	$12–25	Aranda-Díaz et al. (2025)
Pf-SMARRT	Illumina	340	565	-	Sadler et al. (2024)
NOMADS8/16	ONT	1400	292	$25	de Cesare et al. (2024)
NOMADS-MVP	ONT	206	172	$20.92	This study

Supplementary Table 3: Comparison of costs and complexity of NGS protocols for *P. falciparum* genomic surveillance. 'Incubation Time' gives total incubation time across the entire protocol, not including hands-on pipetting time. For pipetting steps, we counted the number of steps from extracted DNA to sequencing for a batch of 48 samples, assuming access to an 8-channel pipette and that master mixes are pre-aliquoted into strip-tubes to minimize 96-well plate pipetting. Qubit-related steps were excluded as they are often optional and/or not done for all samples for convenience. One pipetting step includes both aspirating and dispensing a reagent or sample. Breakdown of per-sample costs for this study is available in STable 1. For other studies, we used per-sample costs from the indicated reference where available.

Minor Clone, %	Parasitemia	Median WSAF, % (IQR, n)
20	100	16.7 (11.5–24.5, n =231)
20	1,000	20.3 (16.4–24.2, n =231)
20	10,000	18.9 (15.9–23.1, n =231)
10	100	12.3 (5.4–18.5, n =231)
10	1,000	10.3 (8.1–13.6, n =231)
10	10,000	10.3 (8.0–12.5, n =230)
5	100	2.5 (0.0–7.8, n =231)
5	1,000	5.5 (4.0–7.0, n =231)
5	10,000	5.5 (4.2–6.7, n =231)
2.5	100	0.2 (0.0–5.0, n =230)
2.5	1,000	2.2 (1.3–4.2, n =231)
2.5	10,000	2.7 (1.9–3.8, n =227)

Supplementary Table 4: Confirming accurate creation of mock DBS samples. Table summarises the median within-sample allele frequency (WSAF) across all expected heterozygous SNPs, in all mock DBS samples that contained mixtures of two laboratory strains. Note how at 1,000 and 10,000 parasites/μL the median WSAF is close to the minor clone proportion; which is expected, if the individual laboratory strains (3D7, Dd2 and HB3) were combined accurately. See Supplementary Note 2 for comments about the 100 parasites/μL mock DBS samples.

Accuracy, % (TP/TN/FP/FN)

Samples with ≥1,000 parasites/μL					
Marker	Clonal	20%	10%	5%	2.5%
crt K76T	100.0 (6/18/0/0)	100.0 (12/6/0/0)	100.0 (12/6/0/0)	100.0 (12/6/0/0)	72.2 (7/6/0/5)
dhfr N51I	100.0 (6/18/0/0)	100.0 (12/6/0/0)	100.0 (12/6/0/0)	100.0 (12/6/0/0)	72.2 (7/6/0/5)
dhfr C59R	100.0 (6/18/0/0)	100.0 (12/6/0/0)	100.0 (12/6/0/0)	100.0 (12/6/0/0)	55.6 (4/6/0/8)
dhfr S108N	100.0 (12/12/0/0)	100.0 (18/0/0/0)	100.0 (18/0/0/0)	100.0 (18/0/0/0)	94.4 (17/0/0/1)
dhps S436F	100.0 (6/18/0/0)	100.0 (12/6/0/0)	100.0 (12/6/0/0)	100.0 (12/6/0/0)	94.4 (11/6/0/1)
dhps A437G	100.0 (12/12/0/0)	100.0 (18/0/0/0)	100.0 (18/0/0/0)	100.0 (18/0/0/0)	88.9 (16/0/0/2)
dhps A613S	100.0 (6/18/0/0)	100.0 (12/6/0/0)	100.0 (12/6/0/0)	100.0 (12/6/0/0)	77.8 (8/6/0/4)
kelch13 R539T	100.0 (2/22/0/0)	-	-	-	-
kelch13 I543T	100.0 (2/22/0/0)	-	-	-	-
kelch13 C580Y	100.0 (2/22/0/0)	-	-	-	-
mdr1 N86Y/F	100.0 (6/18/0/0)	100.0 (12/6/0/0)	94.4 (11/6/0/1)	77.8 (8/6/0/4)	44.4 (2/6/0/10)
mdr1 Y184F	100.0 (6/18/0/0)	100.0 (12/6/0/0)	100.0 (12/6/0/0)	100.0 (12/6/0/0)	88.9 (10/6/0/2)
mdr1 N1042D	100.0 (6/18/0/0)	100.0 (12/6/0/0)	100.0 (12/6/0/0)	100.0 (12/6/0/0)	88.9 (10/6/0/2)
Samples with 100 parasites/μL					
Marker	Clonal	20%	10%	5%	2.5%
crt K76T	100.0 (3/6/0/0)	100.0 (6/3/0/0)	100.0 (6/3/0/0)	77.8 (4/3/0/2)	66.7 (3/3/0/3)
dhfr N51I	100.0 (3/6/0/0)	100.0 (6/3/0/0)	100.0 (6/3/0/0)	55.6 (2/3/0/4)	55.6 (2/3/0/4)
dhfr C59R	100.0 (3/6/0/0)	100.0 (6/3/0/0)	100.0 (6/3/0/0)	55.6 (2/3/0/4)	55.6 (2/3/0/4)
dhfr S108N	100.0 (6/3/0/0)	100.0 (9/0/0/0)	100.0 (9/0/0/0)	66.7 (6/0/0/3)	77.8 (7/0/0/2)
dhps S436F	100.0 (3/6/0/0)	100.0 (6/3/0/0)	88.9 (5/3/0/1)	44.4 (1/3/0/5)	55.6 (2/3/0/4)
dhps A437G	100.0 (6/3/0/0)	100.0 (9/0/0/0)	88.9 (8/0/0/1)	55.6 (5/0/0/4)	55.6 (5/0/0/4)
dhps A613S	100.0 (3/6/0/0)	100.0 (6/3/0/0)	88.9 (5/3/0/1)	33.3 (0/3/0/6)	55.6 (2/3/0/4)
mdr1 N86Y/F	100.0 (3/6/0/0)	88.9 (5/3/0/1)	55.6 (2/3/0/4)	66.7 (3/3/0/3)	33.3 (0/3/0/6)
mdr1 Y184F	100.0 (3/6/0/0)	100.0 (6/3/0/0)	100.0 (6/3/0/0)	77.8 (4/3/0/2)	66.7 (3/3/0/3)
mdr1 N1042D	100.0 (3/6/0/0)	100.0 (6/3/0/0)	100.0 (6/3/0/0)	88.9 (5/3/0/1)	88.9 (5/3/0/1)

Supplementary Table 5: Evaluating the accuracy of antimalarial resistance marker identification across mock samples. Rows display antimalarial resistance markers and columns group samples by minor clone frequency. Each cell shows the accuracy with which the marker was detected across samples and in the parentheses the count of true-positives (TP), true-negatives (TN), false-positives (FP), and false-negatives (FN). Accuracy is defined as $(TP + TN)/(TP + TN + FP + FN)$. Mock samples with 10,000 parasites/μL and 1,000 parasites/μL are grouped at top; and mock samples with 100 parasites/μL are shown at bottom. Only markers were at least one mock sample carries the mutation are shown. Note that the Cambodian strains carrying the *kelch13* markers were only prepared as clonal mock samples, due to the difficulties of making polyclonal samples accurately from DNA.

		Mean Coverage		
Strain	Parasitemia	*hrp2*	*hrp3*	Other genes
3D7	100	984	2263	551
3D7	1000	1968	3716	2113
3D7	10000	2340	4910	3468
Dd2	100	1	2321	661
Dd2	1000	2	4975	1993
Dd2	10000	7	3683	2100
HB3	100	1566	2	716
HB3	1000	1879	3	1645
HB3	10000	2210	9	2481
P.f.−	0	1	13	16

Supplementary Table 6: Mean coverage over *hrp2* and *hrp3* for mock DBS samples. For each strain and parasitemia level, the mean coverage from four sequencing runs is shown for *hrp2*, *hrp3*, and the other eight amplicons in NOMADS-MVP. *P.f.*−, Mock *P. falciparum* negative sample containing only human DNA.

3 Supplementary Figures

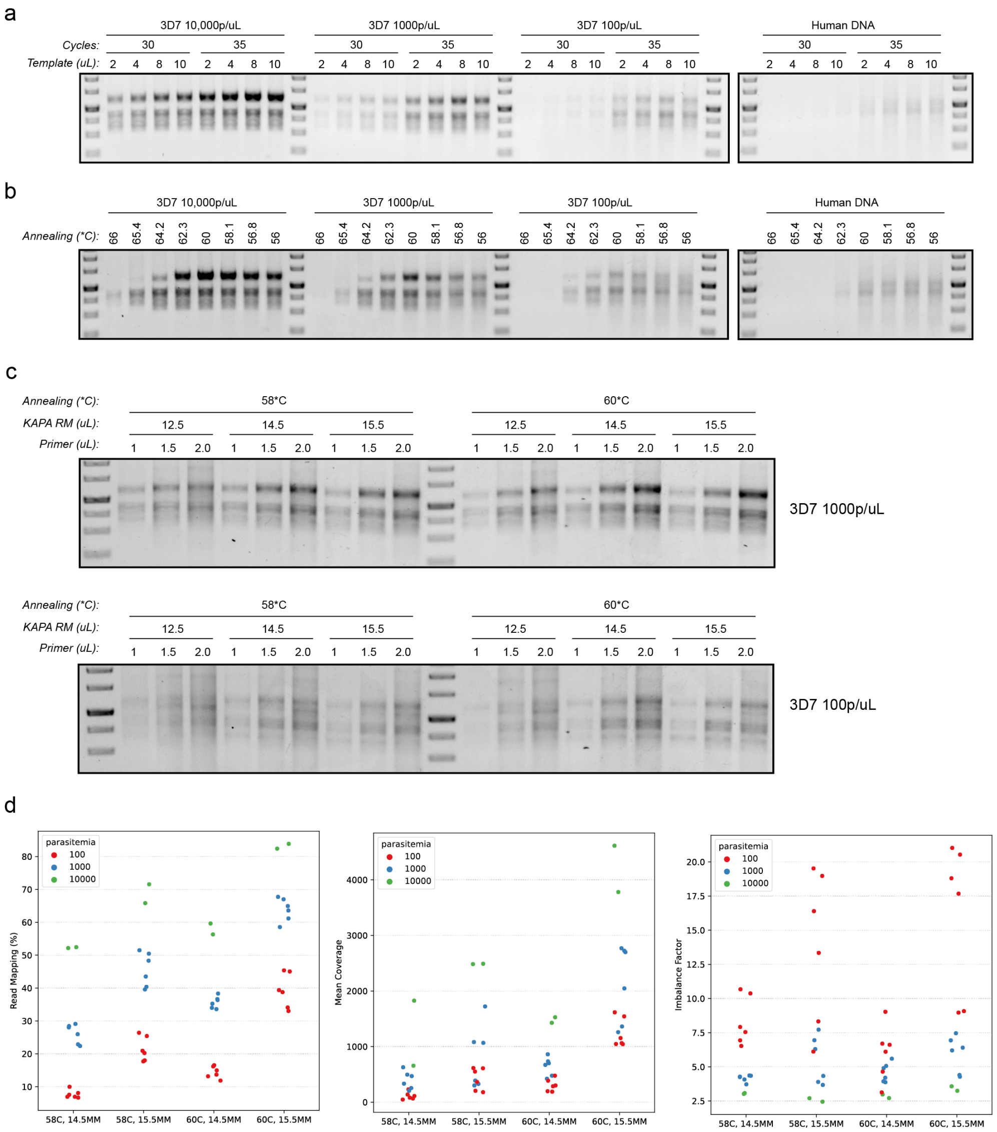

Supplementary Figure 1: Optimising multiplex PCR conditions for NOMADS-MVP using mock samples. (a) Varying number of PCR cycles and template DNA amount. Total reaction volume was fixed at 25 μL. Based on these results, we used 8 μL template DNA and 35-cycles for experiments in panels (b) and (c). (b) Temperature gradient for the annealing/extension step. We restricted to a two-step PCR program with a denaturation step (98°C, 30 seconds) and a combined annealing and extension step (56–66°C temperature gradient, 3 mins). (c) Full factorial experiment varying annealing temperature (58°C, 60°C), KAPA HiFi ReadyMix volume (12.5, 14.5, 15.5 μL) and primer amount (1, 1.5, 2.5 μL) for two different mock samples (3D7 at 1000 parasites/μL and 100 parasites/μL). The KAPA HiFi ReadyMix is a 2X formulation so 12.5 μL is the manufacturer recommended concentration for a 25 μL reaction. Here, we saw boosted sensitivity at 15.5 μL without increased background. (d) Sequencing summary statistics for most promising conditions from (c) across several mock samples at different parasitemia.

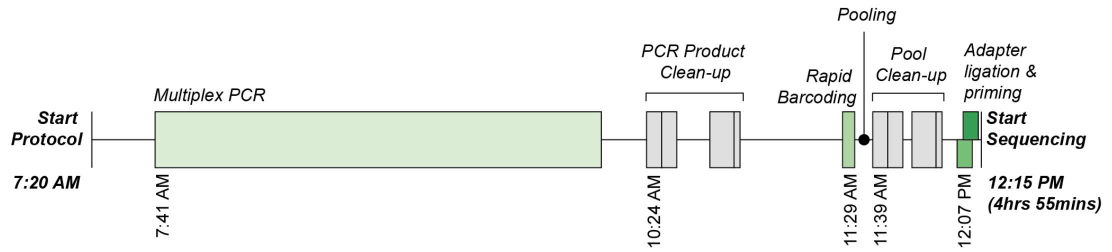

Supplementary Figure 2: Diagram of NOMADS-MVP laboratory protocol timeline. The diagram shows protocol time for a batch of 48 mock DBS samples sequenced at the Max Planck Institute for Infection Biology. Each rectangular box delineates an incubation step, and the lines connecting them were occupied with hands-on laboratory manipulation. Major steps are annotated. Each DNA clean-up step (grey) has four incubations: DNA binding to the AMPure XP magnetic beads; beads binding to the magnet; DNA elution from magnetic beads; removing beads from elute. We generated the timeline by keeping record of the start time of the experiment, the start time of each incubation step, and the start time for sequencing. The majority of the hands-on time occurs after PCR product clean-up and before rapid barcoding and is associated with running an agarose gel and quantifying DNA concentration using the Qubit Flourometer.

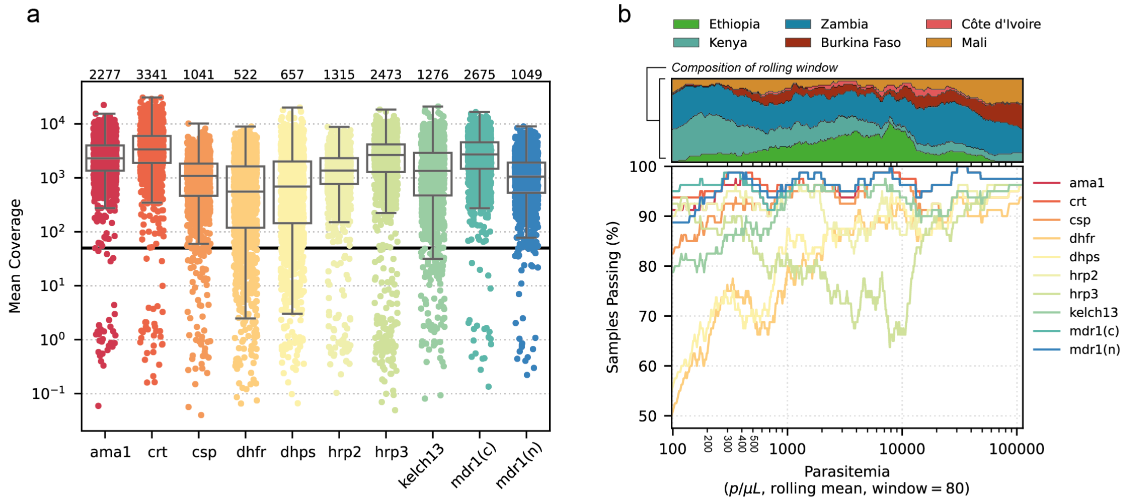

Supplementary Figure 3: Performance of individual NOMADS-MVP amplicons. (a) Boxplots showing mean coverage by amplicon across a total of 1283 DBS samples (1154 field, 129 mock) processed with NOMADS-MVP. The median abundance for each amplicon across all samples is annotated above. *crt* and *ama1* have the highest abundance and are the shortest amplicons. (b) Relationship between parasitemia and samples passing (%) grouped by amplicon. Field samples were ordered from lowest to highest parasitemia, and with a window size of 80 samples a rolling mean of the parasitemia (parasites/μL) was computed (x-axis) and the percentage of samples with $\geq 50\times$ coverage for each amplicon (y-axis) was computed to construct the lines. At lower parasitemias the pass rate of *dhfr* and *dhps* tends to decline. The dip in *hrp3* around 10,000 parasites/μL is due to a large proportion of samples from Ethiopia carrying *hrp3* deletions. The top pane displays what proportion (y-axis) of the 80 samples were from each country (by color) across the rolling windows (x-axis). Only field samples were included.

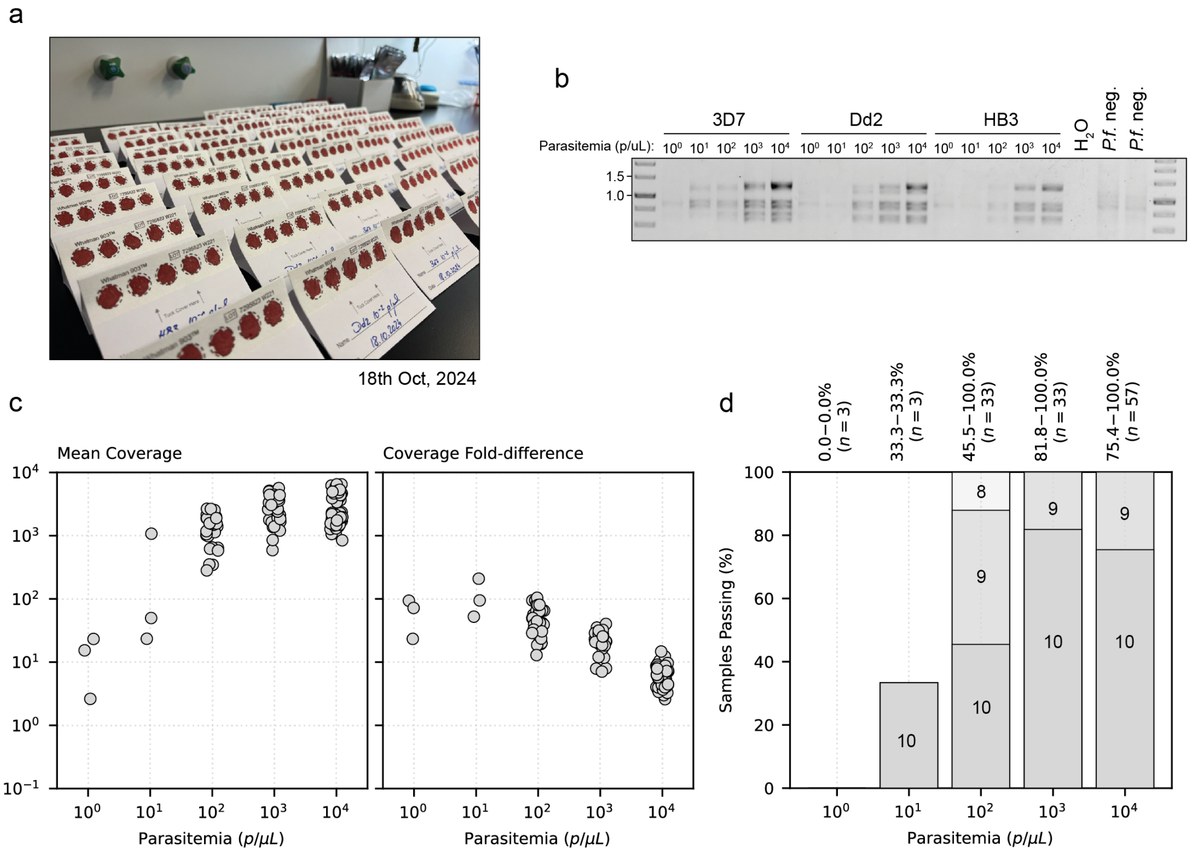

Supplementary Figure 4: Performance of NOMADS-MVP on mock samples. Mock DBS samples were created by culturing laboratory strains of *P. falciparum* and mixing them with human whole blood at different parasitemias. (a) Photo of the mock DBS generated for this manuscript. Each filter paper contains 5 spots of the same mock sample. (b) Agarose gel image of NOMADS-MVP performance on clonal mock DBS samples from 3D7, Dd2 and HB3 ranging from 1 to 10,000 parasites/μL. Negative controls of only water and human DNA are also included. (c) Scatterplots of mean coverage across and coverage fold-difference (Methods) for all mock DBS samples sequenced. (d) Barplot of percentage of mock samples passing across parasitemia. Note that 100% of mock DBS at 100 parasites/μL had at least 8 amplicons with $\geq 50\times$ coverage.

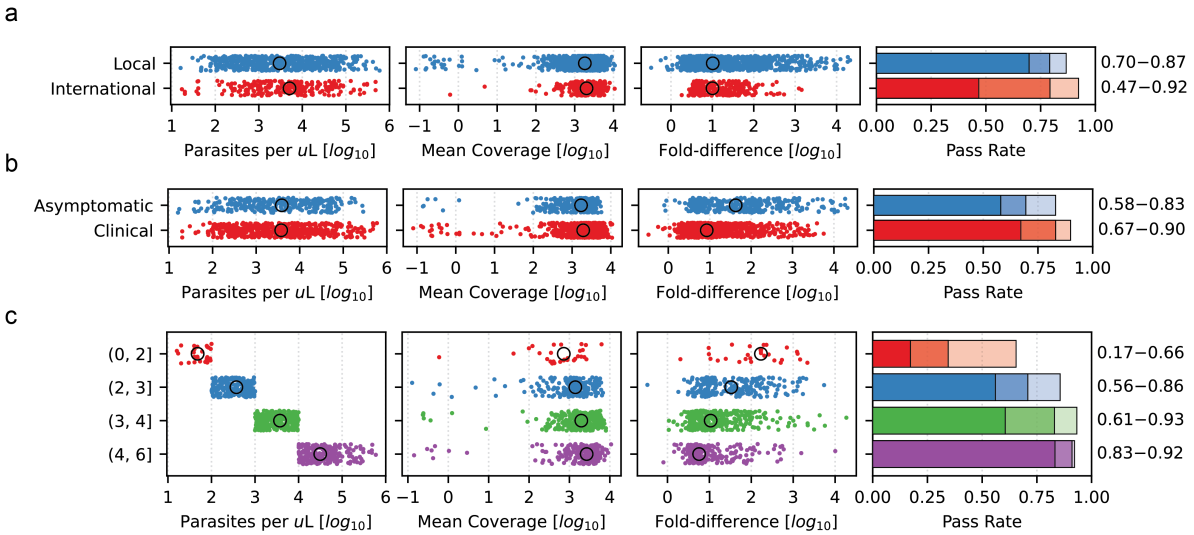

Supplementary Figure 5: Sequencing coverage as a function of sequencing location, clinical status and parasitemia. (a) Effect of sequencing location on sequencing coverage. Local samples ($n = 893$) were sequenced in their country of origin; international samples ($n = 261$, from Mali and Ethiopia) at the Max Planck Institute of Infection Biology, Berlin, Germany. (b) Effect of clinical status on sequencing coverage. Asymptomatic samples ($n = 331$) were from Kenya ($n = 51$), Mali ($n = 42$), and Zambia ($n = 238$); clinical samples ($n = 823$) were collected in all countries. (c) Effect of parasitemia on sequencing coverage. Samples were binned by parasitemia on a logarithmic scale (y-axis). For each panel, each point represents a sample and black circles group medians medians. The left subpanel shows sample parasitemia (parasites/μL); the middle subpanel shows mean per-sample coverage across amplicons; and the right subpanel shows per-sample coverage fold-difference between the least and most abundant amplicon (Methods). All x-axes are on a logarithmic scale. The bar plots show, for each group, the fraction of samples with $> 50\times$ coverage for 8 amplicons (lightest shade), 9 amplicons (intermediate), or all 10 amplicons (darkest); bar height indicates the total fraction of samples with $> 50\times$ coverage for ≥ 8 amplicons. At right, fractions for 10 amplicons and ≥ 8 amplicons passing $> 50\times$ coverage are annotated.

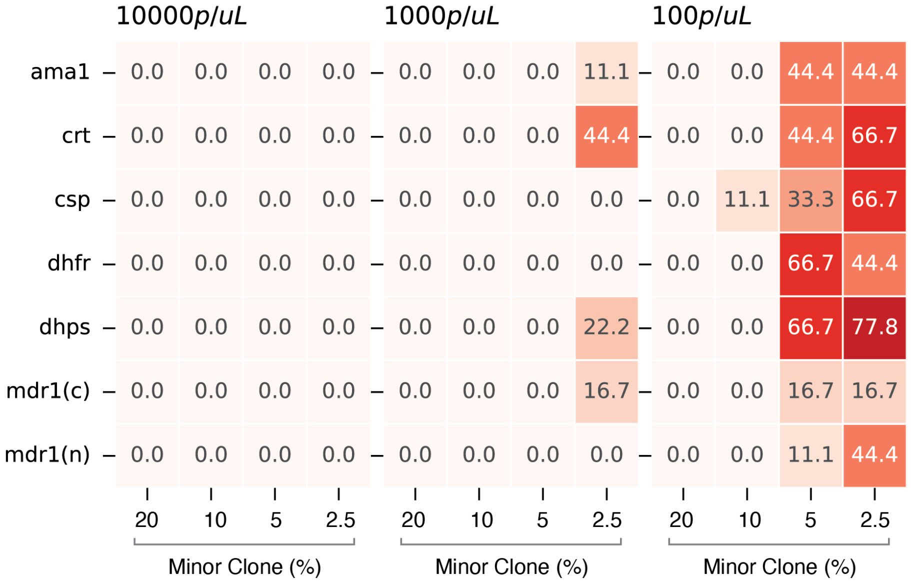

Supplementary Figure 6: Assessing the loss of haplotype signal at low parasitemia levels and minor clone proportions. Heatmap showing the percentage (%) of samples in which the minor clone haplotype generated no within-sample alternative allele (WSAF) signal in the sequencing reads. The data were grouped by parasitemia level (subpanels), amplicon (y-axis), and minor clone proportion (x-axis). For each amplicon and laboratory stain mixture, we determined the set of expected (true) heterozygous SNPs for the minor clone; if greater than 90% of these SNPs had a WSAF less than 0.5% or greater than 99.5%, we classified the haplotype as generating no signal. Notice that at 100 parasites/μL, a considerable fraction of samples generate no signal for the minor clone haplotype across all amplicons. Only amplicons with true heterozygous SNPs were included in the analysis.

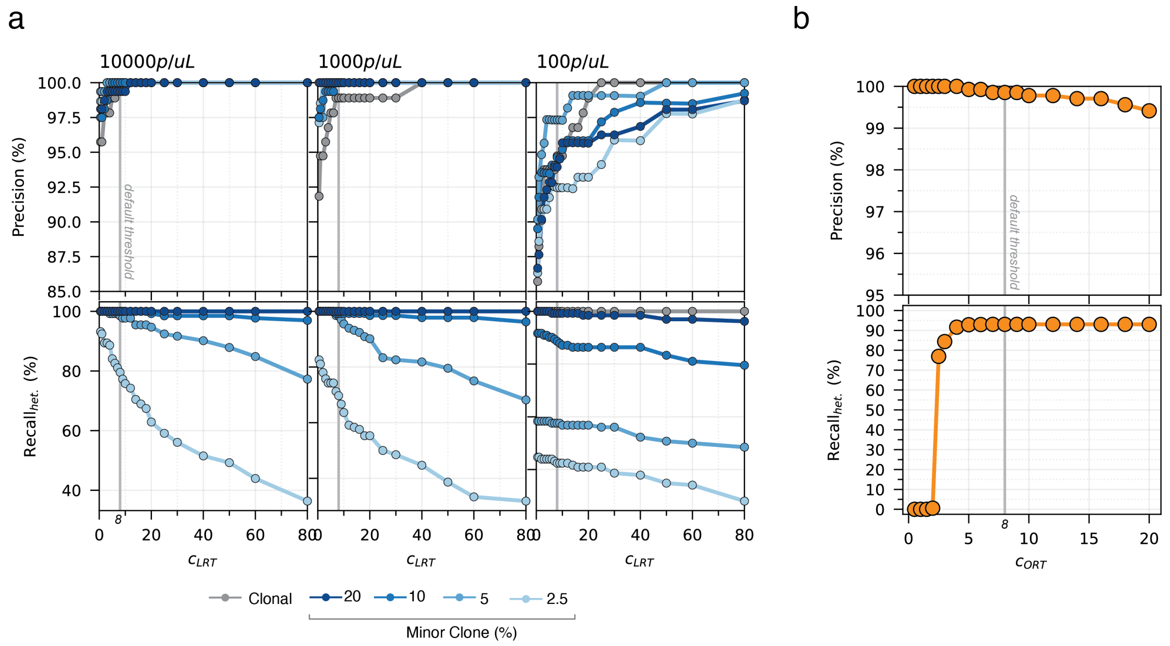

Supplementary Figure 7: Parameter sensitivity analysis of SNP calling performance for *Delve*. Evaluation of SNP calling performance for *Delve* across for 45 mock samples sequenced in triplicate (as in Fig. 4 of main text), assessing sensitivity to variation in two model parameters, with other parameters held constant. (a) Performance as a function of variation in the likelihood-ratio test threshold parameter, c_{LRT}. Top row of subpanels show the SNP calling precision and the bottom show the recall of true heterozygous SNPs (Recall$_{het}$) for different parasitemia levels (subpanel columns) and minor clone proportions (line color). The x-axis shows c_{LRT} value of *Delve*, with the default value, $c_{LRT} = 8$ indicated with a vertical line. Note that increasing the c_{LRT} increases precision in 100 parasites/μL samples, but decreases recall. (b) Peformance as a function of variation in the Strand-bias Odds Ratio Test threshold, c_{ORT}. All samples were pooled for this evaluation as the strand-bias is not influenced by parasitemia or minor clone proportion. Across all parameter values for both statistics, the recall of homozygous alternative SNPs was perfect and is not plotted. See Table 2 of the main text for more details on parameters.

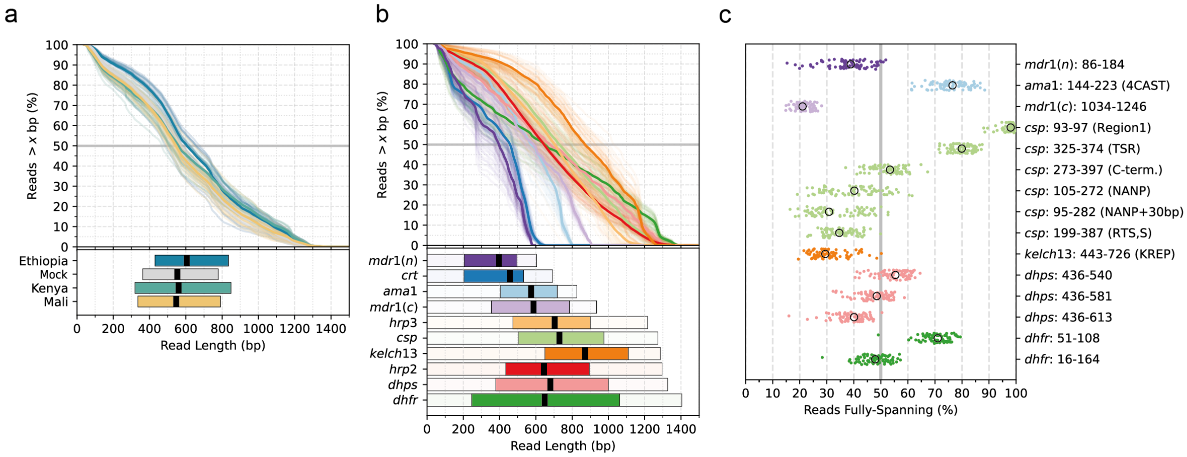

Supplementary Figure 8: Read lengths generated by the NOMADS-MVP protocol. (a) Read length distributions for 80 samples from four countries. The top subpanel shows the percentage of reads (y-axis) longer than a given length (x-axis). Each line represents one sample; colours indicate country. The bottom subpanel shows box plots of read length distributions by country. Boxes indicate the interquartile range, black lines mark the median. (b) Same as (a), but reads are grouped by target amplicon rather by than sample. In the bottom subpanel, the transparent bar behind each box indicates the full amplicon length. (c) Strip plot showing the percentage of sequencing reads fully spanning a given genomic region of interest for each sample. Genomic regions are defined by gene and codon range. 4CAST, region of *ama1* amplified in LaVerriere et al. (2022); TSR, thrombospondin-like type I repeat; KREP, Kelch-repeat propeller. Black circles indicate the region-level medians.

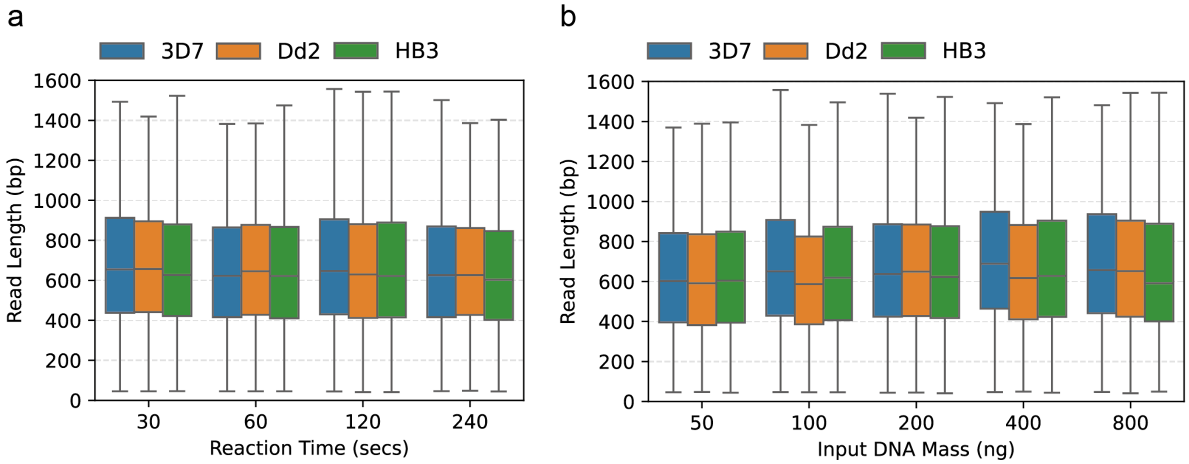

Supplementary Figure 9: Evaluating the influence of the barcoding reaction on read lengths. Rapid Barcoding Kit (SQK-RBK114.96) from ONT fragments reads during barcoding and we evaluated whether modulating either the (a) reaction time or (b) input DNA mass influenced read lengths (y-axis). To isolate the effect of the tagmentation reaction, multiple NOMADS-MVP multiplex PCRs were pooled for 3D7, Dd2 and HB3 at 10,000 parasites/μL. Aliquots of these pools were then used to conduct rapid barcoding for different reaction times or input DNA mass (ng) amounts. The recommended reaction from ONT uses 200ng of input DNA and a 120 second incubation. We observe little effect on read length when these are varied.

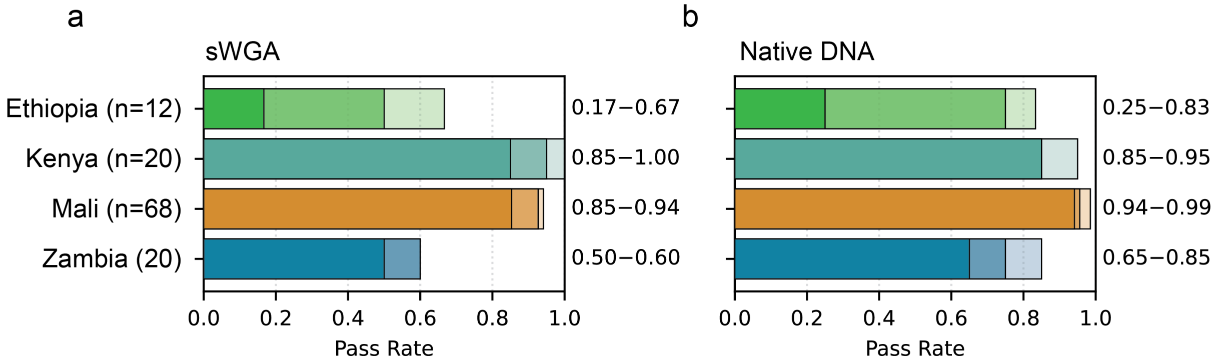

Supplementary Figure 10: Evaluating pass rate on field samples sequenced with and without sWGA. A total of $n = 120$ field samples from four countries were sequenced twice, once with and once without sWGA. (a) Pass rates for samples sequenced where sWGA was peformed on extracted DNA, before multiplex PCR. (b) Pass rates for samples where multiplex PCR was peformed directly on extracted DNA. The bar plots show, for each group, the fraction of samples with $> 50\times$ coverage for 8 amplicons (lightest shade), 9 amplicons (intermediate), or all 10 amplicons (darkest); bar height indicates the total fraction of samples with $> 50\times$ coverage for ≥ 8 amplicons. At right, fractions for 10 amplicons and ≥ 8 amplicons passing $> 50\times$ coverage are annotated. sWGA resulted in no consistent improvement that might justify the additional costs and laboratory steps; in fact, for Ethiopia, Mali and Zambia the pass rates were slighty higher without sWGA.

References

Andrés Aranda-Díaz, Eric Neubauer Vickers, Kathryn Murie, Brian Palmer, Nicholas Hathaway, Inna Gerlovina, Simone Boene, Manuel García-Ulloa, Pau Cisteró, Thomas Katairo, Francis Ddumba Semakuba, Bienvenu Nsengimaana, Hazel Gwarinda, Carla García-Fernández, William Louie, Endashaw Esayas, Clemente Da Silva, Debayan Datta, Shahiid Kiyaga, Innocent Wiringilimaana, Sindew Mekasha Feleke, Adam Bennett, Jennifer L. Smith, Endalamaw Gadisa, Jonathan B. Parr, Melissa D. Conrad, Jaishree Raman, Stephen Tukwasibwe, Isaac Ssewanyana, Eduard Rovira-Vallbona, Cristina M. Tato, Jessica Briggs, Alfredo Mayor, and Bryan Greenhouse. Sensitive and modular amplicon sequencing of Plasmodium falciparum diversity and resistance for research and public health. *Scientific Reports*, 15(1):10737, March 2025. ISSN 2045-2322. doi: 10.1038/s41598-025-94716-5.

Mariateresa de Cesare, Mulenga Mwenda, Anna E. Jeffreys, Jacob Chirwa, Chris Drakeley, Kammerle Schneider, Brenda Mambwe, Karolina Glanz, Christina Ntalla, Manuela Carrasquilla, Silvia Portugal, Robert J. Verity, Jeffrey A. Bailey, Isaac Ghinai, George B. Busby, Busiku Hamainza, Moonga Hawela, Daniel J. Bridges, and Jason A. Hendry. Flexible and cost-effective genomic surveillance of P. falciparum malaria with targeted nanopore sequencing. *Nature Communications*, 15(1):1413, February 2024. ISSN 2041-1723. doi: 10.1038/s41467-024-45688-z.

Emily LaVerriere, Philipp Schwabl, Manuela Carrasquilla, Aimee R. Taylor, Zachary M. Johnson, Meg Shieh, Ruchit Panchal, Timothy J. Straub, Rebecca Kuzma, Sean Watson, Caroline O. Buckee, Carolina M. Andrade, Silvia Portugal, Peter D. Crompton, Boubacar Traore, Julian C. Rayner, Vladimir Corredor, Kashana James, Horace Cox, Angela M. Early, Bronwyn L. MacInnis, and Daniel E. Neafsey. Design and implementation of multiplexed amplicon sequencing panels to serve genomic epidemiology of infectious disease: A malaria case study. *Molecular Ecology Resources*, 22(6):2285–2303, 2022. ISSN 1755-0998. doi: 10.1111/1755-0998.13622.

Jacob M. Sadler, Alfred Simkin, Valery P. K. Tchuenkam, Isabela Gerdes Gyuricza, Abebe A. Fola, Kevin Wamae, Ashenafi Assefa, Karamoko Niaré, Kyaw Thwai, Samuel J. White, William J. Moss, Rhoel R. Dinglasan, Sandrine Nsango, Christopher B. Tume, Jonathan B. Parr, Innocent Mbulli Ali, Jeffrey A. Bailey, and Jonathan J. Juliano. Application of a new highly multiplexed amplicon sequencing tool to evaluate Plasmodium falciparum antimalarial resistance and relatedness in individual and pooled samples from Dschang, Cameroon. *medRxiv*, October 2024. doi: 10.1101/2024.10.03.24314715. URL `https://www.medrxiv.org/content/10.1101/2024.10.03.24314715v2`. Pages: 2024.10.03.24314715.
