# Supplementary material for: Continental-scale genomic surveillance of Plasmodium falciparum malaria across sub-Saharan Africa with rapid nanopore sequencing
Source: Nat Commun. 2026 May 11;17:4218. doi: 10.1038/s41467-026-72358-z (PMC13161212; doi:10.1038/s41467-026-72358-z)
Supplement: Supplementary file 3 — Reporting Summary [file 41467_2026_72358_MOESM3_ESM.pdf]

nature portfolio

Corresponding author(s): Jason A. Hendry

Last updated by author(s): 24/03/2026

Reporting Summary

Nature Portfolio wishes to improve the reproducibility of the work that we publish. This form provides structure for consistency and transparency in reporting. For further information on Nature Portfolio policies, see our Editorial Policies and the Editorial Policy Checklist.

Statistics

For all statistical analyses, confirm that the following items are present in the figure legend, table legend, main text, or Methods section.

n/a	Confirmed	
☐	☒	The exact sample size (*n*) for each experimental group/condition, given as a discrete number and unit of measurement
☐	☒	A statement on whether measurements were taken from distinct samples or whether the same sample was measured repeatedly
☐	☒	The statistical test(s) used AND whether they are one- or two-sided<br>*Only common tests should be described solely by name; describe more complex techniques in the Methods section.*
☐	☒	A description of all covariates tested
☐	☒	A description of any assumptions or corrections, such as tests of normality and adjustment for multiple comparisons
☐	☒	A full description of the statistical parameters including central tendency (e.g. means) or other basic estimates (e.g. regression coefficient) AND variation (e.g. standard deviation) or associated estimates of uncertainty (e.g. confidence intervals)
☐	☒	For null hypothesis testing, the test statistic (e.g. *F*, *t*, *r*) with confidence intervals, effect sizes, degrees of freedom and *P* value noted<br>*Give P values as exact values whenever suitable.*
☐	☒	For Bayesian analysis, information on the choice of priors and Markov chain Monte Carlo settings
☒	☐	For hierarchical and complex designs, identification of the appropriate level for tests and full reporting of outcomes
☐	☒	Estimates of effect sizes (e.g. Cohen's *d*, Pearson's *r*), indicating how they were calculated

Our web collection on statistics for biologists contains articles on many of the points above.

Software and code

Policy information about availability of computer code

Data collection	POD5 and FASTQ files were generated using MinKNOW (v25) from Oxford Nanopore Technologies.
Data analysis	FASTQ files were processed with Nomadic (v0.5.0; https://github.com/JasonAHendry/nomadic). Nomadic maps reads using minimap2 (v2.28), summarizes the output of mapping and coverage of the target amplicons using samtools (v1.17) and bedtools (v1.20), performs variant calling with bcftools call (v1.17) or Delve (v0.1.0; https://github.com/berndbohmeier/delve), and annotates variants using bcftools csq (v1.17). Nomadic uses Python >=v3.10 and a variety of packages included in the standard library (pandas, numpy). The dashboard was implemented using the Dash library from plotly (v2.17.1).

For manuscripts utilizing custom algorithms or software that are central to the research but not yet described in published literature, software must be made available to editors and reviewers. We strongly encourage code deposition in a community repository (e.g. GitHub). See the Nature Portfolio guidelines for submitting code & software for further information.

Data

Policy information about availability of data

All manuscripts must include a data availability statement. This statement should provide the following information, where applicable:

- Accession codes, unique identifiers, or web links for publicly available datasets
- A description of any restrictions on data availability
- For clinical datasets or third party data, please ensure that the statement adheres to our policy

The raw sequencing data from mock DBS samples have been deposited in the \href{https://www.ncbi.nlm.nih.gov/sra}{NCBI Sequence Read Archive} under the

BioProject accession code \href{https://www.ncbi.nlm.nih.gov/bioproject/?term=(PRJNA1401910)}{PRJNA1401910}. The raw sequencing data from field DBS samples are not publicly available due to ethical, legal, and data governance restrictions associated with the countries of origin, but may be made available by request to the corresponding author and subject to approval of the relevant national authorities. A response to requests is typically provided within 4 -- 6 weeks. Access, if granted, will be provided for the duration and under the terms specified in the applicable data access or transfer agreements.

All other data supporting the findings of the paper are available in the Source Data and Supplementary Information.

Research involving human participants, their data, or biological material

Policy information about studies with human participants or human data. See also policy information about sex, gender (identity/presentation), and sexual orientation and race, ethnicity and racism.

Reporting on sex and gender	Sex and gender were not relevant to any analyses conducted and therefore are not reported.
Reporting on race, ethnicity, or other socially relevant groupings	No data was collected on race, ethnicity or socially relevant groupings.
Population characteristics	No population characteristics are reported as, aside from patient country, no individual-level patient data was used in analyses.
Recruitment	Patients were recruited either passively through attendance at a primary health facility for a symptomatic illness (Senegal; Burkina Faso; Cote d'Ivoire; Zambia, Gen8; Ethiopia) or actively as part of cross-sectional (Kenya; Zambia, MIS2024) or longitudinal (Mali) studies.
Ethics oversight	All blood samples were collected from patients with P. falciparum malaria, with informed consent from the patient or from a parent or guardian. In all cases, consent allowed for the samples to be used for purposes such as this study. This study was conducted using the collected samples only; there was no human subject contact.<br><br>All studies received ethical approval from an appropriate research ethics committee: in Senegal, ethical approval for the SEN19/49 study was granted by the Comité National d'Ethique pour la Recherche en Santé (000317/MSAS/CNERS/SP); in Torodo, Mali, ethical approval for a cohort study in 2022 was granted by the Ethics Committee of Charité (EA2/264/21) and the Faculty of Medicine, Pharmacy and Odontostomatology (FMPOS) at the University of Bamako (N°2022/20/CE/USTTB/24.01.22); in Burkina Faso, ethical approval for the AMTIP study was granted by the Comité d'Ethique Institutionnel / Institut National de Santé Publique (2023-10/MSHP/SG/INSP/CEI); in Côte d'Ivoire, ethical approval for the study "Surveillance génomique de Plasmodium falciparum aux CTA à Bouaké, Côte d'ivoire" was granted by the Direction Médicale et Scientifique du aux Centre Hospitalier et Universitaire (192MSHPCMU/CHU-B/DG/DMS/ONAR/24) and by the Ethikkomission der Charité Universitätsmedizin (EA2/171/24); in Nigeria, ethical approval for samples collected in 2023--2024 was granted by the Ethical Committee of Ladoke Akintola University of Technology Teaching Hospital, Ogbomoso (LTH/OGB/EC/2022/304); in Zambia, ethical approval for the 2024 Malaria Indicator Survey (MIS2024) and the 2024--2025 \textit{hrp2/3} Surveillance Study was granted by the Research Ethics Committee at the University of Zambia (5055-20241) and the Tropical Diseases Research Centre Ethics Review Committee (TRC/C4//03/2024), respectively; in Kenya, ethical approval for samples collected in the ATSB study was granted by the KEMRI Scientific and Ethics Review Unit (KEMRI/SERU/CGHR/368/4189; CDC Project ID 0900f3eb81d7ec3c and 0900f3eb82546323); in Ethiopia, ethical approval for the ARSUNA study was granted by the Arsi University institutional review board (AU/HSC/ST-129/5494) and the Federal Democratic Republic of Ethiopia, Ministry of Education (17/256/476/24).

Note that full information on the approval of the study protocol must also be provided in the manuscript.

Field-specific reporting

Please select the one below that is the best fit for your research. If you are not sure, read the appropriate sections before making your selection.

[x] Life sciences [ ] Behavioural & social sciences [ ] Ecological, evolutionary & environmental sciences

For a reference copy of the document with all sections, see nature.com/documents/nr-reporting-summary-flat.pdf

Life sciences study design

All studies must disclose on these points even when the disclosure is negative.

Sample size	As part of the study we processed field samples from Senegal (n=110), Mali (n=176), Cote d'Ivoire (n=29), Burkina Faso (n=88), Nigeria (n=71), Zambia (n=477), Kenya (n=290) and Ethiopia (n=163); as well as mock samples made from laboratory strains in Berlin (n=213). For the mock samples, we selected a sample size and set (e.g. variety of laboratory strains and combinations) that would be sufficient to validate key aspects of our sequencing approach, and performed sequencing in triplicate.
Data exclusions	From Figure 2 onwards, samples processed with sWGA were excluded (121/1,404, 8.6%). After initial testing with sWGA, we determined that the step did not improve sequencing performance, so from August 2024 we stopped including it in our sequencing procedure. As it is no longer part of our protocol and is not being used, the results using sWGA are not relevant to an assessment of our protocol's performance. This exclusion and the reasons for it are described in the Main Text and Methods of the manuscript.
Replication	All field samples were sequenced only once. For mock samples, we sequenced samples in replicate in two different validation analyses:

1. For the hrp2/3 analysis, 9 positive controls (3D7, Dd2, HB3 at 10000, 1000 and 100p/uL) and 3 negative controls were sequenced in quadruplicate.
2. For the minor clone sensitivity analysis, 45 positive controls (described in the Methods) and 3 negative controls were sequenced in triplicate.

Randomization	Randomisation was not relevant to our manuscript analyses. Regardless, for most experiments, samples from a study were randomly selected for sequencing to minimise any batch effects. In other cases, samples were selected for sequencing runs based on convenience or availability.
Blinding	Sample blinding was not required for the analyses.

Reporting for specific materials, systems and methods

We require information from authors about some types of materials, experimental systems and methods used in many studies. Here, indicate whether each material, system or method listed is relevant to your study. If you are not sure if a list item applies to your research, read the appropriate section before selecting a response.

Materials & experimental systems

n/a	Involved in the study
☒	☐ Antibodies
☒	☐ Eukaryotic cell lines
☒	☐ Palaeontology and archaeology
☒	☐ Animals and other organisms
☒	☐ Clinical data
☒	☐ Dual use research of concern
☒	☐ Plants

Methods

n/a	Involved in the study
☒	☐ ChIP-seq
☒	☐ Flow cytometry
☒	☐ MRI-based neuroimaging

Plants

Seed stocks	*Report on the source of all seed stocks or other plant material used. If applicable, state the seed stock centre and catalogue number. If plant specimens were collected from the field, describe the collection location, date and sampling procedures.*
Novel plant genotypes	*Describe the methods by which all novel plant genotypes were produced. This includes those generated by transgenic approaches, gene editing, chemical/radiation-based mutagenesis and hybridization. For transgenic lines, describe the transformation method, the number of independent lines analyzed and the generation upon which experiments were performed. For gene-edited lines, describe the editor used, the endogenous sequence targeted for editing, the targeting guide RNA sequence (if applicable) and how the editor was applied.*
Authentication	*Describe any authentication procedures for each seed stock used or novel genotype generated. Describe any experiments used to assess the effect of a mutation and, where applicable, how potential secondary effects (e.g. second site T-DNA insertions, mosiacism, off-target gene editing) were examined.*
